# The association between serum HDL levels and infertility among American women aged 20–44 years: A retrospective cross-sectional study of NHANES, 2013–2020

**DOI:** 10.1371/journal.pone.0311618

**Published:** 2024-10-07

**Authors:** Hui Wang, Dongmei Wang, Hui Chen, Liping Yang, Chunying Xie, Zhenzhen Ruan, Zhe Han

**Affiliations:** Center for Reproductive Medicine, Yantaishan Hospital, Yantai, Shandong, China; Hamadan University of Medical Sciences, School of Public Health, ISLAMIC REPUBLIC OF IRAN

## Abstract

**Background:**

Infertility is a significant national public health concern, and the World Health Organization (WHO) predicts that it will rank as the third most prevalent disease following tumors, cardiovascular and cerebrovascular diseases. The impact of dysfunctional lipoproteins on female infertility remains relatively understudied; therefore, the research focuses on exploring the relationship between serum high-density lipoprotein (HDL) concentration and infertility.

**Methods:**

This is a retrospective cross-sectional study where we employed multivariate logistic regression analysis to examine the association between serum HDL concentrations and female infertility. The strength of association was quantified using odds ratios (OR) along with their corresponding 95% confidence intervals and statistical significance was evaluated at a level of P < 0.05 (two-tailed).

**Results:**

The study found that there was a significant correlation between serum HDL and infertility without adjusting the model (OR = 0.62, 95%CI 0.48–0.82, P<0.001). After adjusting for covariates, a weak correlation between HDL and infertility remained (OR = 0.70, 95%CI 0.49–1.00). When HDL concentrations were divided into quartiles, there was a trend of strengthened correlation between HDL and infertility risk with the increase in HDL concentrations. Specifically, individuals in the highest concentration quartile exhibited a 44.0% lower risk of infertility compared to those in the lowest concentration quartile (95% CI 0.38–0.84). In the age-stratified analysis, after adjusting for covariates, the correlation between HDL and infertility was statistically insignificant across all age groups. Furthermore, after categorizing HDL levels into quartiles, we observed a dose-dependent trend between HDL and the reduction of female infertility risk in the adjusted models of the secondary infertility group. Specifically, in the adjusted model, the high-concentration group exhibited a 67.0% lower risk of infertility compared to the low-concentration group (OR = 0.33; 95% CI: 0.12–0.940, P = 0.04).

**Conclusion:**

Our research findings suggest weak negative correlation between serum HDL and female infertility. However, upon stratified analysis by age, the correlation between HDL and infertility did not attain statistical significance. In cases of secondary infertility, a subtle dose-dependent trend was observed between serum HDL and infertility.

## Introduction

Infertility is a multifactorial reproductive disorder that poses a significant challenge to the reproductive health of couples during their childbearing years, exerting a profound impact on family well-being [1–3]. According to the World Health Organization (WHO), infertility is defined as the failure of women engaging in regular unprotected sexual intercourse to achieve pregnancy within a period of 12 months. Clinically, primary infertility refers to the absence of any previous pregnancy history, while secondary infertility pertains to individuals with prior pregnancies [4].

High-density lipoprotein (HDL), traditionally regarded as the "good cholesterol," has garnered widespread recognition for its roles in reverse cholesterol transport, antioxidation, anti-inflammation, and protection of vascular endothelial cells [5,6]. Recent studies have suggested that abnormalities in the structure, abundance, or function of HDL may be intimately linked to female fertility. For instance, multiple studies conducted on women undergoing in vitro assisted reproductive technology (ART) have shown that the antioxidant function of HDL in follicular fluid has a certain role in reducing oocyte fragmentation, improving oocyte quality, and maintaining normal fertilization of oocytes, and the results of ART treatment are also related to the HDL levels in women [7–10]. However, contrasting findings also exist. For example, a research team from Houston Methodist demonstrated that reducing cholesterol levels in infertile mice using bacterial protein serum opacity factor (SOF) restored their fertility [11]. A Mendelian randomization study, on the other hand, indicated no significant association between HDL levels and the risk of female infertility [12].

Collectively, the relationship between serum HDL and infertility represents a complex yet intriguing area of research. By delving deeper into the specific roles of HDL within the female reproductive system and its potential associations with infertility, we may uncover novel therapeutic strategies and diagnostic tools for patients struggling with infertility.

## Methods

### Study population

**Fig 1:**
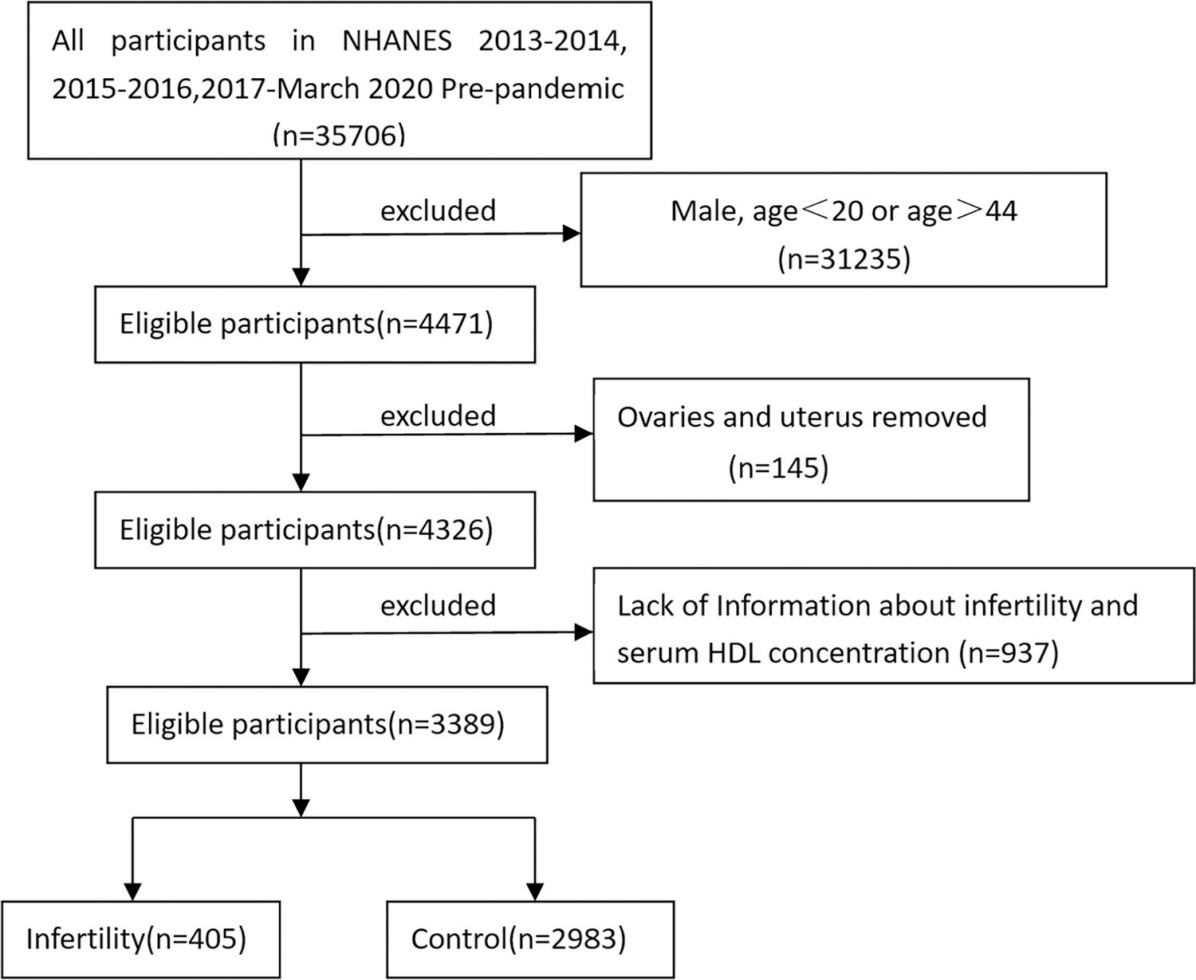
Flow chart of sample selection.

The NHANES database, a comprehensive survey conducted by the National Center for Health Statistics, offers extensive health and nutrition information regarding the general population of the United States. We collected reproductive health data from female participants aged 20–44 years between 2013 and 2020. The NHANES dataset is publicly accessible on www.cdc.gov/nchs/nhanes/. All participants provided their written informed consent, and the NCHS Ethics Review Board granted ethical approval for the conduct of NHANES activities. The NHANES database is publicly accessible and anonymous, and our research was a secondary analysis of the data contained within, thus rendering it impossible for us to identify any participants during or after the data collection process. Our study included 3,389 women after excluding those without biochemical data or a history of hysterectomy or bilateral oophorectomy (Fig 1). The raw data were provided in the supplementary materials.

### Research variable

Infertility, as a study variable, was assessed through the reproductive health section of the NHANES questionnaire. Skilled examiners administered the questions in a mobile examination center (MEC), utilizing a computer-assisted personal interview (CAPI) system during the interview conducted at the MEC. Publicly available data on the NHANES website includes measurements of HDL serum concentration and other study variables such as age, race, marital status, education level, poverty to income ratio, BMI, smoking habits, alcohol consumption patterns, and biochemical indicators including albumin alkaline phosphatase levels, cholesterol levels, triglyceride levels and creatinine levels.

### Statistical analysis

We employed two multiple logistic regression models: Model one without any adjusted variables, and Model two with adjusted variables. In Model two, we screened all variables from the previous study. If the regression coefficient of a covariate on infertility was P<0.1 or significantly correlated with infertility, it was considered as a potential confounding factor and adjusted accordingly.

In the description of the study population, continuous variables following a normal distribution are represented by the mean value accompanied by the standard deviation. Alternatively, if they do not follow a normal distribution, they are represented by the median value along with the 25th and 75th percentiles. Categorical variables are expressed as percentages.

To better elucidate the relationship between HDL and infertility, we additionally performed a quartile grouping analysis on the continuous variable of HDL [13,14]. To account for the potential impact of age on infertility, we conducted a hierarchical analysis considering age as a covariate. Furthermore, recognizing the possibility of differential effects on HDL in primary and secondary infertility cases, we performed a stratified analysis. The strength of association was quantified using odds ratios (OR) along with their corresponding 95% confidence intervals and statistical significance was evaluated at a level of P < 0.05 (two-tailed).

The statistical analyses were performed utilizing R statistics packages (http://www.R-project.org, The R Foundation) and Empower Stats (http://www.empowerstats.com, X&Y Solutions, Inc, Boston, MA). Statistical significance was evaluated at a level of P < 0.05 (two-tailed).

## Result

### Demographic profile of the study participants

A total of 3389 women aged 20-44years were enrolled in this study, including 405 individuals diagnosed with infertility (11.95%, Fig 1). The characteristics of infertility group and control group are presented in Table 1. Compared to the group without infertility, infertile women were older (34.07 VS.31.79) and a higher BMI (32.03 VS. 29.42). They were more likely to be drinkers (9.38% VS 6.13%), to be smokers (34.07% VS.28.08%), to have pelvic infection (8.64% VS. 4.32%) and take female hormones (3.95% VS. 2.38%). There were no statistical differences in race and education level.

**Table 1:** The characteristics of study infertility group and control group.

	Infertility(n = 405)	Control(n = 2984)	P-value
**Age **	34.07± 6.57	31.79 ± 7.24	<0.001
**Race**			0.91
Mexican American	67 (16.54%)	503 (16.86%)	
Other Hispanic	40 (9.88%)	326 (10.93%)	
Non-Hispanic White	138 (34.07%)	945 (31.67%)	
Non-Hispanic Black	95 (23.46%)	686 (22.99%)	
Non-Hispanic Asian	46 (11.36%)	375 (12.57%)	
Other race	19 (4.69%)	149 (4.99%)	
**BMI**	32.03± 9.17	29.42 ± 8.39	<0.001
**RIP**	2.527 ± 1.66	2.31 ± 1.60	0.01
**Education level**			0.65
Less than 9th grade	16 (3.95%)	153 (5.13%)	
9-11th grade	50 (12.35%)	301 (10.09%)	
High school graduate	75 (18.52%)	577 (19.34%)	
Some college or AA degree	156 (38.52%)	1117 (37.43%)	
College graduate or above	108 (26.67%)	835 (27.98%)	
NA	0 (0.000%)	1 (0.03%)	
**Alcohol drinking**			0.03
Yes	38 (9.38%)	183 (6.13%)	
No	314 (77.53%)	2334 (78.22%)	
NA	53 (13.09%)	467 (15.65%)	
**Smoking**			0.04
Yes	138 (34.07%)	838 (28.08%)	
No	267 (65.93%)	2144 (71.85%)	
Don’t know	0 (0.00%)	2 (0.07%)	
**Regular periods**			0.18
Yes	384 (94.82%)	2776 (93.03%)	
No	21 (5.19%)	208 (6.97%)	
**Ever been pregnant**			<0.001
Yes	332 (81.98%)	2104 (70.51%)	
No	72 (17.78%)	879 (29.46%)	
Don’t know	1 (0.25%)	1 (0.03%)	
**Marital status**			<0.001
Yes	125 (30.86%)	669 (22.42%)	
No	17 (4.20%)	103 (3.45%)	
Never married	263 (64.94%)	2212 (74.13%)	
**Pelvic infection**			<0.001
Yes	35 (8.64%)	129 (4.32%)	
No	367 (90.62%)	2837 (95.07%)	
NA	3 (0.74%)	18 (0.60%)	
**Female hormones taken**			0.09
Yes	16 (3.95%)	71 (2.38%)	
No	388 (95.80%)	2910 (97.52%)	
NA	1 (0.25%)	3 (0.10%)	

BMI: Body Mass Index; RIP: Ratio of family income to poverty; NA: Not Available.

Alcohol drinking: Ever have 4/5 or more drinks every day.

Smoking: Smoked at least 100 cigarettes in life.

### Differences in serum biochemical indices between infertile patients and the control group

As depicted in the Table 2, the rank sum test revealed a significant disparity in serum biochemistry between the infertility group and the control group, with notably lower levels of HDL observed in the infertility group compared to the control group(1.40 VS. 1.47).Although we studied the relationship between HDL and infertility in this paper, other biochemical data, such as Alkaline Phosphatase (ALP), Alanine Aminotransferase (ALT),Gamma Glutamyl Transferase(GGT),and Triglycerides also showed a possible correlation with infertility. However, when models were analyzed to remove confounding factors, there was no statistical significance between these variables and infertility.

**Table 2:** Differences in serum biochemical indices between infertile patients and the control group.

	Infertility(n = 405)	Control(n = 2984)	P-value
ALP(IU/L)	69.37 ± 48.22	65.07 ± 22.07	<0.01
AST(U/L)	20.95 ± 12.83	20.48 ± 12.16	0.47
ALT	20.21± 14.03	18.70± 14.40	<0.01
Toal calcium(mmol/L)	2.30 ± 0.09	2.31 ± 0.09	0.27
Cholesterol(mmol/L)	4.75 ± 0.99	4.67± 0.92	0.06
CPK(IU/L)	115.67 ± 135.16	113.57 ± 125.00	0.35
GGT(IU/L)	25.82 ± 45.39	20.37 ± 27.52	<0.001
LDH (IU/L)	129.01 ± 27.72	129.93 ± 29.39	0.55
Toal protein(g/dL)	7.09 ± 0.46	7.13 ± 0.45	0.05
Albumin(g/dL)	4.04 ± 0.42	4.10 ± 0.38	<0.01
Creatinine(umol/L)	62.24 ± 17.42	62.84 ± 25.56	0.65
Globulin(g/dL)	3.05 ± 0.43	3.03 ± 0.417	0.30
Triglycerides(mmol/L)	1.47 ± 1.01	1.28 ± 0.87	<0.001
HDL (mmol/L)	1.37 ± 0.42	1.47 ± 0.41	<0.001

ALP: Alkaline Phosphatase (IU/L) AST: Aspartate Aminotransferase.

ALT: Alanine Aminotransferase (U/L) CPK: Creatine Phosphokinase.

GGT: Gamma Glutamyl Transferase LDH: Lactate Dehydrogenase.

HDL: High-density lipoprotein.

### Association between serum levels of HDL and female infertility

The relationship between HDL and female infertility risk is illustrated in Table 3. In the unadjusted model, patients with low HDL concentrations exhibited a higher risk of infertility (OR = 0.62, 95%CI 0.48–0.82, P<0.001). After screening and adjusting for 25 covariates, the correlation between HDL and the risk of female infertility still show a slight statistical difference(OR = 0.70, 95%CI 0.49–1.00, P<0.05). Covariates were incorporated based on previous descriptions or clinical expertise [15]. When HDL concentrations were divided into quartiles, a significant negative correlation between HDL levels and the risk of female infertility was observed in the unadjusted model. In the adjusted model, a weak correlation between HDL and infertility remained, and there was a trend of strengthened correlation between HDL and infertility risk with the increase in HDL concentrations. Specifically, individuals in the highest concentration quartile exhibited a 44.0% lower risk of infertility compared to those in the lowest concentration quartile (95% CI 0.38–0.84).

**Table 3:** Association between serum levels of HDL (mmol/L) and female infertility.

	Odds Ratio(95%CI) P-value	
	Non-adjusted model (n = 3389)	Adjust model (n = 3068)
HDL	0.62 (0.48, 0.82) <0.001	0.70 (0.49, 1.00) 0.048
Q1	1	1
Q2	0.70 (0.52, 0.92) 0.012	0.80 (0.58, 1.10) 0.17
Q3	0.62 (0.47, 0.82) <0.001	0.78 (0.56, 1.09) 0.15
Q4	0.50 (0.37, 0.68) <0.001	0.56 (0.38, 0.84) <0.01

Non-adjusted model adjusted for: None.

Adjust model adjust for: Covariates listed in Tables 1 and 2, except HDL.

Q1:0.41–1.13 Q2:1.14–2.26 Q3:2.27–3.39 Q4:3.39–4.11.

To demonstrate the disparity in infertility risk associated with HDL levels across different age groups, we categorized participants into five distinct age cohorts, as depicted in Table 4. In the unadjusted model group, among individuals aged under 25(OR = 0.22 95% CI 0.08–0.64). and those aged 25-30(OR = 0.23 95% CI 0.11–0.46), a significant negative correlation was observed between HDL concentration levels and the risk of infertility. However, after adjusting for covariates, the statistical significance was no longer present. Furthermore, In the adjusted model group, when stratifying HDL into four classes based on different age groups, among individuals over 40 years old, the second-highest HDL concentration group exhibited a statistically significant negative correlation with the risk of infertility compared to the low-concentration group (OR = 0.43 95% CI 0.21–0.89).

**Table 4:** Age subgroup analysis for association between serum levels of HDL (mmol/L) and female infertility.

	Age <25	Age ≥25, <30	Age ≥30, <35	Age ≥35, <40	Age ≥40
	Odds Ratio (95%CI) P-value				
HDL (Non-adjusted model)	0.22 (0.08, 0.64) 0.02	0.23 (0.11, 0.46) <0.001	0.75 (0.43, 1.34) 0.33	0.77 (0.46, 1.27) 0.30	1.03(0.64, 1.65) 0.92
Q1	1	1	1	1	1
Q2	0.40 (0.18, 0.90) 0.03	0.95 (0.50, 1.81) 0.88	0.54 (0.28, 1.03) 0.06	0.82 (0.45, 1.49) 0.52	0.87(0.49, 1.55) 0.64
Q3	0.29 (0.12, 0.68) 0.005	0.50 (0.26, 0.97) 0.04	0.70 (0.39, 1.28) 0.25	0.94 (0.55, 1.61) 0.82	0.66 (0.37, 1.21) 0.18
Q4	0.26 (0.09, 0.71) 0.009	0.25(0.11, 0.55) <0.001	0.56 (0.29, 1.08) 0.08	0.59(0.320, 1.081) 0.09	0.82 (0.46, 1.45) 0.49
HDL (Adjust model)	0.43 (0.11, 1.61) 0.21	0.40 (0.15, 1.05) 0.06	1.11(0.50, 2.47) 0.80	1.00 (0.48, 2.12) 0.99	0.59(0.30, 1.17) 0.13
Q1	1	1	1	1	1
Q2	0.52 (0.21, 1.34) 0.18	1.46 (0.69, 3.09) 0.32	0.76 (0.34, 1.64) 0.49	1.28(0.64, 2.56) 0.49	0.62 (0.32, 1.20) 0.15
Q3	0.45 (0.16, 1.23) 0.12	0.94 (0.41, 2.16) 0.89	1.34(0.63, 2.86) 0.44	1.25 (0.61, 2.57) 0.54	0.43 (0.21, 0.89) 0.02
Q4	0.40 (0.10, 1.53) 0.18	0.48 (0.17, 1.37) 0.17	1.04(0.42, 2.57) 0.93	0.76 (0.32, 1.77) 0.52	-

Non-adjusted model adjust for: None.

Adjust model adjust for: Covariates listed in Tables 1 and 2, except age and HDL.

Q1:0.41–1.13 Q2:1.14–2.26 Q3:2.27–3.39 Q4:3.39–4.11.

### Association between serum HDL levels and the risk of primary and secondary infertility

Taking into account the variations in infertility factors, we conducted separate analyses for primary and secondary infertility, as depicted in Table 5. The results indicated that after adjusting for covariates, the concentration level of HDL consistently demonstrated a mitigating effect on the risks of both primary and secondary infertility, albeit not reaching statistical significance, the associations were robust. Specifically, in the primary infertility group, an increment of one unit in HDL levels corresponded to a 23% reduced risk of infertility, while in the secondary infertility group, each unit increase in HDL translated to a 56% lowered risk of infertility. Furthermore, upon stratifying HDL levels into four distinct categories, we observed a dose-dependent relationship between HDL and the reduction of female infertility risk in adjusted models of secondary infertility group. Specifically, in the adjusted model, the high concentration group exhibited a 67.0% lower risk of infertility compared to the low concentration group (OR = 0.33 95% CI 0.12–0.940,P = 0.04).

**Table 5:** Association between serum levels of HDL (mmol/L) and the risk of primary and secondary infertility.

	Non-adjusted model		Adjust model	
	OR (95%CI) P value		OR (95%CI) P value	
	Primary infertility	Secondary infertility	Primary infertility	Secondary infertility
HDL	0.81 (0.61, 1.08) 0.16	0.22 (0.11, 0.43) <0.001	0.77 (0.52, 1.14) 0.19	0.44 (0.17, 1.12) 0.09
Q1	1	1.00	1.00	1
Q2	0.71 (0.52, 0.98) 0.039	0.64 (0.35, 1.19) 0.16	0.79 (0.55, 1.12) 0.19	0.87(0.41, 1.84) 0.71
Q3	0.77 (0.56, 1.05) 0.10	0.30 (0.16, 0.58) <0.001	0.85(0.59, 1.23) 0.30	0.45 (0.19, 1.05) 0.06
Q4	0.65(0.47, 0.91) 0.01	0.19(0.09, 0.41) <0.001	0.63 (0.41, 0.97) 0.04	0.33 (0.12, 0.94) 0.04

Non-adjusted model adjust for: None.

Adjust model adjust for: Covariates listed in Tables 1 and 2, except HDL.

Q1:0.41–1.13 Q2:1.14–2.26 Q3:2.27–3.39 Q4:3.39–4.11.

## Discussion

This study is a cross-sectional survey targeting American women aged 20–40, aiming to explore the relationship between serum HDL levels and infertility.

Previous studies have primarily focused on alterations in lipid metabolism among infertile individuals or patients with polycystic ovary syndrome (PCOS) [16,17], and they have also examined the impact of HDL level differences on oocyte fertilization, embryo quality, and clinical pregnancy rates among individuals undergoing assisted reproductive technology (ART) treatments [18,19]. Our study, after adjusting for confounding factors, still revealed a negative correlation between serum HDL concentration and the risk of infertility. In line with previous research, HDL may exert a protective effect on reproductive health through its anti-inflammatory and antioxidant mechanisms. The development of oocytes within the ovaries is regulated by the balance between oxidation and antioxidation [20]. When intracellular reactive oxygen species (ROS) levels elevate, the antioxidant mechanisms become imbalanced. Oxidative stress can lead to the conversion of HDL into dysfunctional HDL, and the total oxidative stress index in serum increases in patients with unexplained infertility [21]. Kadriye Erdoğan et al. utilized the ratio of myeloperoxidase (MPO) to paraoxonase (PON) as a marker for dysfunctional HDL and found an elevated MPO/PON ratio in patients with unexplained infertility, which was associated with a decrease in the number of Grade 1 embryos and the rate of high-quality blastocysts [22].

Inflammatory diseases can alter the composition of follicular fluid and lead to infertility due to reduced oocyte quality. HDL particles possess potent anti-inflammatory properties, and the presence of HDL in follicular fluid may modulate the local inflammatory state within the follicle [23]. A study using fast protein liquid chromatography (FPLC) to isolate HDL revealed that higher anti-inflammatory activity of HDL in follicular fluid is associated with an increased chance of oocytes developing into high-quality embryos [24].

The protective effect of HDL on reproductive health varies with the causes of infertility. With the economic growth, the proportion of secondary infertility has surpassed that of primary infertility [25,26]. Secondary infertility mainly results from reproductive tract infections caused by previous pregnancy and delivery with normal ovarian function, so the age of secondary infertility is often higher than that of primary infertility [27,28]. As age increases, both the quality of oocytes and mitochondrial function decline, leading to an increase in chromosomal segregation errors and the generation of ROS [29–31]. A study has revealed that there is no significant difference in serum HDL levels between patients with Premature ovarian insufffciency (POI) and the healthy control group [32]. Our results indicate that the protective effect of HDL against infertility in secondary infertility exhibits a slightly dose-dependent trend, with a 67.0% lower risk of infertility in the high-concentration group compared to the low-concentration group.

A significant study that deserves mention in the context of exploring the relationship between HDL and infertility found no clear correlation between HDL and the risk of infertility. Possible explanations for the discrepancies in these findings could be attributed to the utilization of distinct databases, varying study populations, and different research methodologies employed in the two studies [12].

Despite the large sample size of this study and adjustments for potential confounding factors, a correlation between HDL levels and infertility was observed. However, due to the small number of infertile individuals, the positive sample size in each age subgroup was relatively low, and the correlation between HDL and infertility did not reach statistical significance in these age subgroups. Therefore, the study results cannot rule out the presence of bias. Additionally, compared to the study population involved in assisted reproductive technology research, although we endeavored to eliminate multiple confounding factors, this study was unable to exclude the influence of male factors on female infertility outcomes.

In summary, our findings suggest that serum HDL may serve as a protective factor against infertility. Although the correlation between HDL and infertility was not statistically significant in the age-stratified analysis, a significant association was observed between high HDL concentrations and infertility, particularly in the context of secondary infertility.

## Supporting information

S1 File Raw data. (XLSX)
